# Assessment of environmental and spatial factors influencing the establishment of *Anopheles gambiae* larval habitats in the malaria endemic province of Woleu-Ntem, northern Gabon

**DOI:** 10.1186/s12936-024-04980-5

**Published:** 2024-05-21

**Authors:** Neil-Michel Longo-Pendy, Silas Lendzele Sevidzem, Boris Kevin Makanga, Saturnin Ndotit-Manguiengha, Stravensky Térence Boussougou-Sambe, Piazzy Obame Ondo Kutomy, Judicaël Obame-Nkoghe, Lynda-Chancelya Nkoghe-Nkoghe, Barclaye Ngossanga, Felicien Kassa Mvoubou, Christophe Roland Zinga Koumba, Ayôla Akim Adegnika, Abdul-Safiou Razack, Jacques François Mavoungou, Rodrigue Mintsa-Nguema

**Affiliations:** 1 Unité de Recherche en Ecologie de la Santé (URES), Centre Interdisciplinaire de Recherches Médicales de Franceville (CIRMF), Franceville, Gabon; 2 Laboratoire d’Ecologie des Maladies Transmissibles (LEMAT), Université Libreville Nord (ULN), Libreville, Gabon; 3 Institut de Recherche en Écologie Tropicale (IRET), Libreville, Gabon; 4 Agence Gabonaise d’Etudes et d’Observations Spatiales (AGEOS), Libreville, Gabon; 5 Centre de Recherches Médicales de Lambaréné, Lambaréné, Gabon; 6 Institut Für Tropenmedizin, Eberhard Karls Universität, Tübingen, Germany; 7 Programme National de Lutte Contre Le Paludisme (PNLP), Libreville, Gabon; 8 Universite Cheikh Anta Diop de Dakar (UCAD), Dakar, Sénégal; 9 Université des Sciences et Techniques de Masuku (USTM), Franceville, Gabon; 10 Institute of Public Health and Hygiene, Libreville, Gabon; 11 Fondation Pour la Recherche Scientifique (FORS), P.O. Box 88, Cotonou, Benin; 12 German Center for Infection Research (DZIF), Partner site Tübingen, Tübingen, Germany; 13 Department of Zoology and Entomology, Faculty of Natural and Agricultural Sciences, University of the Free State, Phuthaditjhaba, Republic of South Africa

**Keywords:** *Anopheles* larval habitats, Spatial distribution, Malaria control, Environmental factors, Gabon

## Abstract

**Background:**

This study aimed to assess the spatial distribution of *Anopheles* mosquito larval habitats and the environmental factors associated with them, as a prerequisite for the implementation of larviciding.

**Methods:**

The study was conducted in December 2021, during the transition period between the end of the short rainy season (September–November) and the short dry season (December-February). Physical, biological, and land cover data were integrated with entomological observations to collect *Anopheles* larvae in three major towns: Mitzic, Oyem, and Bitam, using the "dipping" method during the transition from rainy to dry season. The collected larvae were then reared in a field laboratory established for the study period. After the *Anopheles* mosquitoes had emerged, their species were identified using appropriate morphological taxonomic keys. To determine the influence of environmental factors on the breeding of *Anopheles* mosquitoes, multiple-factor analysis (MFA) and a binomial generalized linear model were used.

**Results:**

According to the study, only 33.1% out of the 284 larval habitats examined were found to be positive for *Anopheles* larvae, which were primarily identified as belonging to the *Anopheles gambiae* complex. The findings of the research suggested that the presence of *An. gambiae* complex larvae in larval habitats was associated with various significant factors such as higher urbanization, the size and type of the larval habitats (pools and puddles), co-occurrence with *Culex* and *Aedes* larvae, hot spots in ambient temperature, moderate rainfall, and land use patterns.

**Conclusions:**

The results of this research mark the initiation of a focused vector control plan that aims to eradicate or lessen the larval habitats of *An. gambiae* mosquitoes in Gabon's Woleu Ntem province. This approach deals with the root causes of malaria transmission through larvae and is consistent with the World Health Organization's (WHO) worldwide objective to decrease malaria prevalence in regions where it is endemic.

## Background

Despite significant progress over the past decade, including the introduction of new artemisinin-based anti-malarial drugs, the use of long-lasting insecticidal nets (LLINs) and indoor residual spraying (IRS), malaria remains a major threat to the health of people in sub-Saharan Africa. Children under the age of 5 account for more than 80% of the victims of this parasitic disease [1–3]. In Gabon, particularly in the northern forest region of Woleu Ntem Province, malaria remains one of the most commonly diagnosed parasitic diseases [4]. With the growing phenomenon of *Anopheles* resistance to insecticides [5, 6] and changes in feeding and biting behaviour undermining malaria control systems [7], the World Health Organization (WHO) is recommending additional measures that can have a direct impact on malaria control such as the rational management of *Anopheles* mosquito larval habitats. Larviciding programmes have received considerable attention in sub-Saharan Africa in recent years [8, 9]. Although some reports suggest that combining LLINs and IRS has a positive impact on the fight against malaria, it is important to note that there are also limitations to this approach. Those limitations are the need for a huge logistics (aerial application) in areas with extensive larval habitats and the lack of knowledge about the larval habitats of some specific *Anopheles* species and the geographical availability of larval habitats in specific areas [10, 11]. Effective implementation of these additional measures in a given environment requires the identification and characterization of *Anopheles* larval habitats as well as the determination of key ecological factors that influence their presence [12]. Landscape features such as topography and land cover/use play an important role in determining the distribution and microclimatic conditions of larval habitats, which in turn affect the distribution of adult *Anopheles* vectors. Human activities, such as road building, infrastructure development, deforestation and agricultural practices, can also affect habitat distribution and stability [13].

Remote sensing is a powerful tool for studying the larval habitats of mosquitoes, particularly those that transmit diseases such as malaria [14, 15]. Several studies have highlighted the increased effectiveness of integrating remote sensing into the control systems of these vector-borne diseases [16]. At the national level, this technology has enabled the identification of regions that are conducive to the formation of larval habitats, including wetlands, mangroves, swamps, rice fields and temporary water bodies [17, 18]. In addition, remote sensing tools are being used to monitor environmental changes that may influence the distribution of mosquito larval habitats [19–22], thereby facilitating the efficient allocation of resources by health authorities in their vector control efforts. Despite its recognized role in the fight against malaria, this approach remains under-utilized by Central African countries.

In Gabon, a country highly affected by malaria [23–25], particularly in the province of Woleu-Ntem [4], research on malaria parasite transmission, particularly the identification of *Anopheles* larval habitats, remains scant. In this context, entomological studies using remote sensing to identify factors favoring *Anopheles* mosquito presence could significantly advance targeted malaria control by eliminating surface waters conducive to mosquito proliferation. In addition, the threat posed by the recent introduction of *Anopheles stephensi* in the Horn of Africa [26–29] and its recent sightings in Nigeria and Ghana [28] warrants the need for extensive larval surveys that includes *Aedes* larval habitats that are known to be favourable sites for *An. stephensi* [30, 31]. The current entomological study in the three main towns of Woleu-Ntem Province was, therefore, designed to characterize the environmental and anthropogenic elements potentially associated with the presence of *Anopheles* larvae in breeding habitats.

## Methods

### Characteristics of the study area

**Fig. 1:**
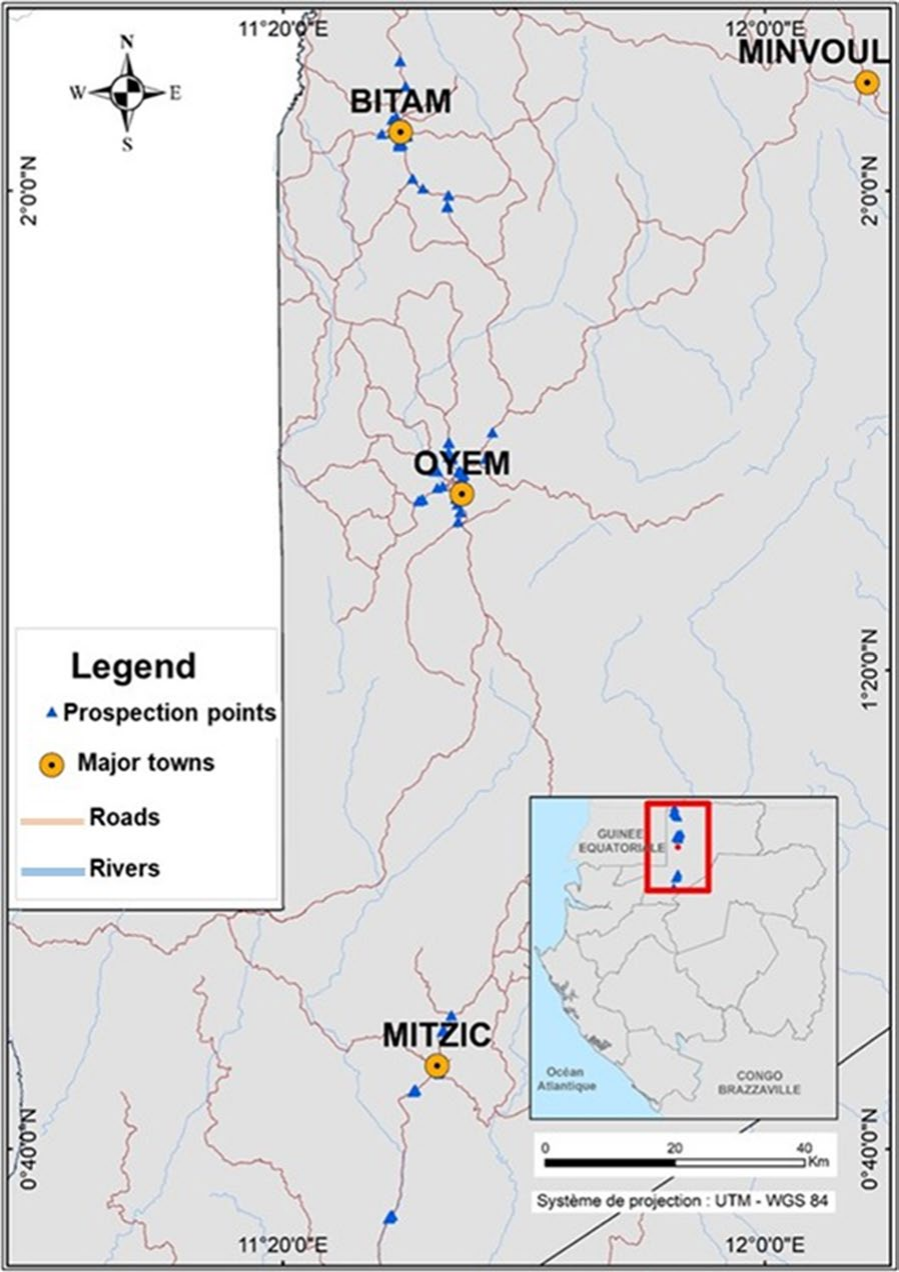
Geolocation of prospection sites in Woleu-Ntem province

A field study was carried out in the three main towns of Woleu Ntem Province during the transition period between the rainy and dry seasons, from 10 to 27 December 2021. Woleu-Ntem is one of the nine provinces of Gabon. Its capital town is Oyem, but other towns also form part of this province, such as Bitam, Minvoul, Mitzic and Médouneu. According to the 2013 census, this province had 155,000 inhabitants. A high proportion of the surface area of this province is covered by the equatorial forest. The present study was conducted in three major towns (Mitzic, Oyem and Bitam) of Woleu-Ntem (Fig. 1 and Table 1). Bitam is the capital of the Ntem department and is located at 2° 05′ 00″ north latitude and 11° 29′ 00″ east longitude. It is located 30 km from the Cameroonian border and not far from Equatorial Guinea. According to the 2013 population census, the town had 27, 923 inhabitants. It is covered by a tropical rainforest with an equatorial climate. The average monthly temperature and relative humidity are 26 °C and 98% respectively (https://www.historique-meteo.net/). The city of Oyem is the capital of the Woleu department, with a population of over 60000. It is located at latitude 1° 37′ 00″ north and longitude 11° 35′ 00″ east. In Gabon, the towns of Bitam and Oyem share borders with Cameroon and Equatorial Guinea. Trade with neighbouring countries such as Equatorial Guinea and Cameroon is common. The vegetation of Oyem is of rainforest type with an equatorial climate. The average monthly temperature and relative humidity are 31 °C and 63%, respectively. The town of Mitzic is the capital of the Okano Department and is 111.47 km from Oyem, the capital of Woleu Ntem. Geographically, it is located between latitude 0° 42′ 11″ north and longitude 11° 33′ 57″ east. According to the 2013 population census, the population of this town was estimated at 8755 inhabitants. The climate of this area is tropical savanna type. The rapidly growing population of Woleu Ntem is experiencing tremendous urban development (mainly the construction of roads, schools, hospitals, public facilities and increased commercial activities), which could potentially lead to an increase in the number and types of malaria vector habitats. However, the peri-urban and village forest ecosystems of this province are experiencing high levels of logging activity by forestry companies, which could distort the natural habitats, species diversity, distribution and aggressiveness of mosquitoes. In addition to the type of activities in the different areas of Woleu-Ntem, it was noted that the type of housing varied from urban (central town) with mostly cement block buildings and few wooden houses to rural (villages) with mostly wooden houses [4].

**Table 1:** Characteristics of the study sites

Country	Locality	Latitude	Longitude	Levels of urbanization	Proportion of positive *Anopheles* breeding sites n (% [CI])	*p-*value
Gabon	Bitam	2.8061111	11.49452778	1	31(68[53–81])	< 0.0001
	Mitzic	0.78411111	11.54911111	2	44(27[20–34])	
	Oyem	1.59900000	11.57602778	3	19(26[17–38])	

Levels of urbanization: 1 = Low, 2 = Medium, 3 = High

### Mosquito larval habitat characterization

Larvae were collected from potential larval habitats in the three main towns of Woleu-Ntem, namely Mitzic, Oyem, and Bitam, for 17 consecutive days. The collection took place daily from 8:00 to 14:00 and a standard dipper, as described by MW Service [32], was used for this purpose. To ensure that we visited all areas of each town to search for potential larval habitats, this collection was conducted with the assistance of locals who are more familiar with the town. And each larval habitat was explored only once. After collecting the *Anopheles* larvae, they were taken back to the insectarium for rearing. When they emerged as adults, each *Anopheles* was identified by their physical characteristics using a standard dichotomous key [33, 34]. An aquatic habitat was considered positive if at least one *Anopheles* spp. larvae was found. Moreover, potential larval habitats were visually inspected in order to determine the different types of larval habitats. Categorical and quantitative variables were analysed to describe the type of larval habitat used by *Anopheles*. The study considered several categorical variables, including the presence or absence of *Culex* and *Aedes* mosquito larvae, pollution linked to organic decomposition, the artificial or natural aspect of the larval habitat, the presence or absence of green algae in the larval habitat, the longevity of the larval habitat (temporary or permanent), and the appearance of the larval habitat (puddle, rut, domestic container, basin, breeze block). For quantitative variables, the diameter and depth of the larval habitat and the distance from the larval habitat to the nearest house were measured. All *Anopheles* larval habitats were georeferenced.

### Vegetation cover index and surface temperature

The study analysed vegetation dynamics by utilizing the Normalized Difference Vegetation Index (NDVI) obtained from MODIS TERRA MOD13Q1 V6.1 satellite imagery. The imagery is derived from the National Oceanic and Atmospheric Administration's (NOAA-AVHRR) advanced high-resolution radiometer, with a spatial resolution of 250 m in December 2021. The syntheses, which were produced every 16 days, were downloaded from https://lpdaac.usgs.gov/products/mod13q1v061/. The study utilized land surface temperature images obtained from the MODIS TERRA MOD21C3.061 sensor, available on the website https://lpdaac.usgs.gov/products/mod21c3v061/ for December 2021. These images are synthesized every 8 days and have a spatial resolution of 1 km. The NDVI and land surface temperature images are at level 3, where they are already corrected and projected into the World Geodetic System 1984 (WGS 84) geographic coordinate system. For each site, the monthly and overall period averages were calculated and presented them as NDVI and temperature maps. Additionally, temperature data were edited to remove any outliers.

### Precipitation and land use

The study used daily precipitation data with a spatial resolution of 0.05° (approximately 5 km) from the CHRIPS (Climate Hazards Group InfraRed Precipitation with Station data) programme, downloaded from https://www.chc.ucsb.edu/data/ chirps for December 2021. The study used daily precipitation data with a spatial resolution of 0.05° (approximately 5 km) from the CHRIPS (Climate Hazards Group InfraRed Precipitation with Station data) program, downloaded from https://www.chc.ucsb.edu/data/ chirps for December 2021. The data was resampled to the resolution of the NDVI data to improve correlation. The monthly accumulation data from three meteorological stations in Gabon, namely Libreville, Mvengué, and Port-Gentil, were used to calibrate the satellite precipitation data. These stations are the most well-supplied with data and the data was obtained from the Gabon Meteorological Directorate. The validation method was based on calculating the correlation coefficient between the two precipitation time series. This procedure was previously used by Arvor et al*.* [35] and Boussougou [36]. The correlation coefficients for the Libreville, Port-Gentil, and Mvengué stations are 0.87, 0.87, and 0.88, respectively, indicating a high compatibility between the two time series at each station. The Pearson correlation test was used to establish the significance of the obtained coefficients, with a confidence level set at 0.05 or 5%. The p-values obtained were equal to 2.2e-16, indicating that the correlations were statistically significant for all selected stations used for validation and as references.

The land use data was obtained by manually digitizing a high-resolution (10 cm) image acquired by drone over the main urban centres of the study area (Oyem, Bitam, and Mitzic) using QGIS software. This enabled the mapping of all structures, including the extraction of buildings, for the month of December 2021.

### Statistical and geospatial analysis

All statistical analyses were performed using R version 4.0.2 statistical software. First, a Multiple Factor Analysis was performed using the "FactoMineR" package, to describe the type of larval habitats used by *Anopheles* from a set of quantitative (diameter and depth of the larval habitat and the distance from the larval habitat to the nearest house) and qualitative variables (pollution linked to organic decomposition, artificial or natural aspect of the larval habitat, presence or absence of green algae in the larval habitat, longevity of the larval habitat, and the appearance of the larval habitat) collected for each larval habitat [37, 38]. To examine their effect on the occurrence of larvae in larval habitats, we specifically excluded the variables of the presence of *Culex* spp. larvae and *Aedes* spp. larvae from the MFA. The occurrence of *Anopheles* larvae in larval habitats was examined, along with different larval habitats characteristics. This was accomplished using the "stats" software package to conduct a generalized linear binomial model analysis [39]. The study analysed the presence/absence of *Anopheles* larvae in larval habitats as the response variable, and the presence/absence of *Culex* mosquito larvae and *Aedes* mosquito larvae, along with MFA dimensions 1 and 2, as explanatory variables. A forward stepwise model-selection procedure based on Akaike Information Criterion (AIC) [40] was used to obtain the most parsimonious models. Although models with a ΔAICC < 2 were retained [41], the model with the lowest AIC was considered the best fit model.

The Google Earth Engine (GEE) platform was used to collect time series of NDVI, temperature, and precipitation images during December 2021, with the aim of calculating monthly averages of surface temperatures and NDVI, as well as the total precipitation for the month. Subsequently, data processing was carried out using mapping software such as QGIS and ArcGIS to create maps and represent building density.

## Results

### Larval habitats characterization

**Fig. 2:**
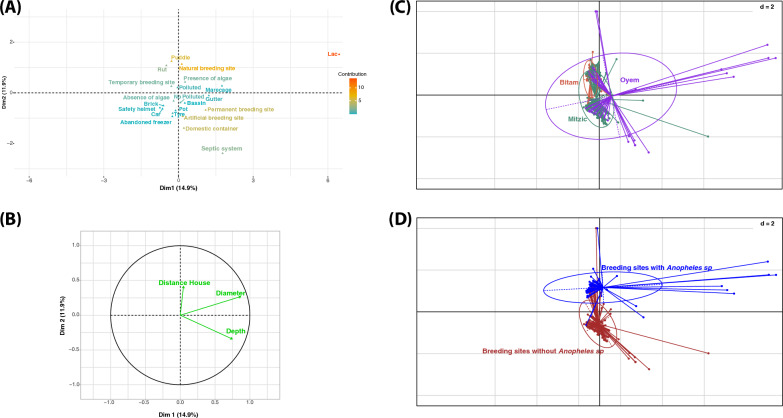
Larval habitat characterization according to physical characteristics. Canonical weight of each variable on the principal dimension: the position of the character for qualitative variables (**A**) and the size of the arrow for quantitative variables (**B**) indicate the importance of the parameter. **C** and **D** MFA diagrams showing correlations between different localities, and between positive *Anopheles* larval habitats and physical characteristics

**Fig. 3:**
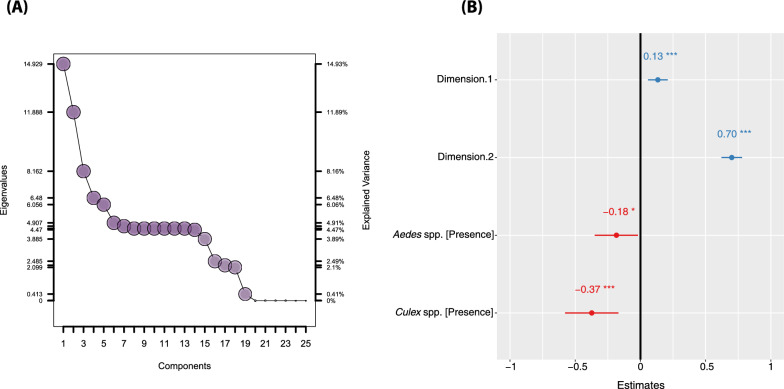
**A** Dimensions selected with the broken stick model. **B** Generalized linear model showing the effect of different larval habitats characteristics that favour *Anopheles* larval occurrence. Legend: 0 ‘***’ 0.001 ‘**’ 0.01 ‘*’ 0.05 ‘.’ 0.1 ‘’ 1; red colour: negative correlation; blue colour: positive correlation

In this study, 284 potential larval habitats were surveyed in the three main towns (Mitzic, Oyem and Bitam) of Woleu Ntem Province in Gabon. A total of 157 positive larval habitats were identified, showing great diversity in the values of physical and environmental variables. The town of Bitam (69%, 31/45) had a significantly high percentage of *Anopheles*-positive larval habitats, followed by the towns of Mitzic (27%, 44/166) and Oyem (26%, 19/73) [(Chi2 = 30.938, *p-*value < 0.0001) (Table 1)]. All mosquitoes of the genus *Anopheles* (1570 specimens) belonged to the *Anopheles gambiae* complex. Multifactorial analysis (MFA) selected the first two dimensions (Dim) using the broken stick model test (Fig. 3A) [42]. These two dimensions accounted for 26.8% of the total variance. Dimension 1 (14.9%) can be interpreted as the size of the larval habitat, characterized by the diameter and depth of the larval habitats (Fig. 2A, B). Dimension 2 (11.9%) can be interpreted as the physiognomy of the larval habitat, characterized by the varying water content of the *Anopheles* larval habitats. Furthermore, the MFA indicates an overlap between the larval habitats in the cities, with the confidence ellipse of Oyem encompassing those of Bitam and Mitzic (Fig. 2C). On the other hand, the MFA analysis enabled us to differentiate between larval habitat used by *Anopheles* (positive larval habitats) and those not used by *Anopheles* (negative larval habitats) (Fig. 2D). The distinction between anopheline-positive and anopheline-negative larval habitats was primarily observed along dimension 2, which refers to the size of the larval habitat, rather than dimension 1, which refers to the physical characteristics of the larval habitat (Fig. 2D).

### Macro-environmental factors affecting the presence of Anopheles gambiae s.l larvae in larval habitats

Our ecological modeling approach identified the first two dimensions (Dim. 1 & Dim. 2), as well as the presence of *Culex* spp and *Aedes* spp larvae, as essential factors in explaining the presence of *Anopheles gambiae *sensu lato (*s.l*.) larvae in the larval habitats. The GLM results show that the size of larval habitats, especially in medium-sized ones like puddles and ruts, significantly affects the occurrence of larvae from the *An. gambiae* complex (Figs. 2A and 3). This occurrence was also negatively correlated with the occurrence of larvae of other mosquito vectors, particularly *Culex* spp. and *Aedes* spp. [(with estimates of −0.37 and −0.18 respectively) (Fig. 3A)].

**Fig. 4:**
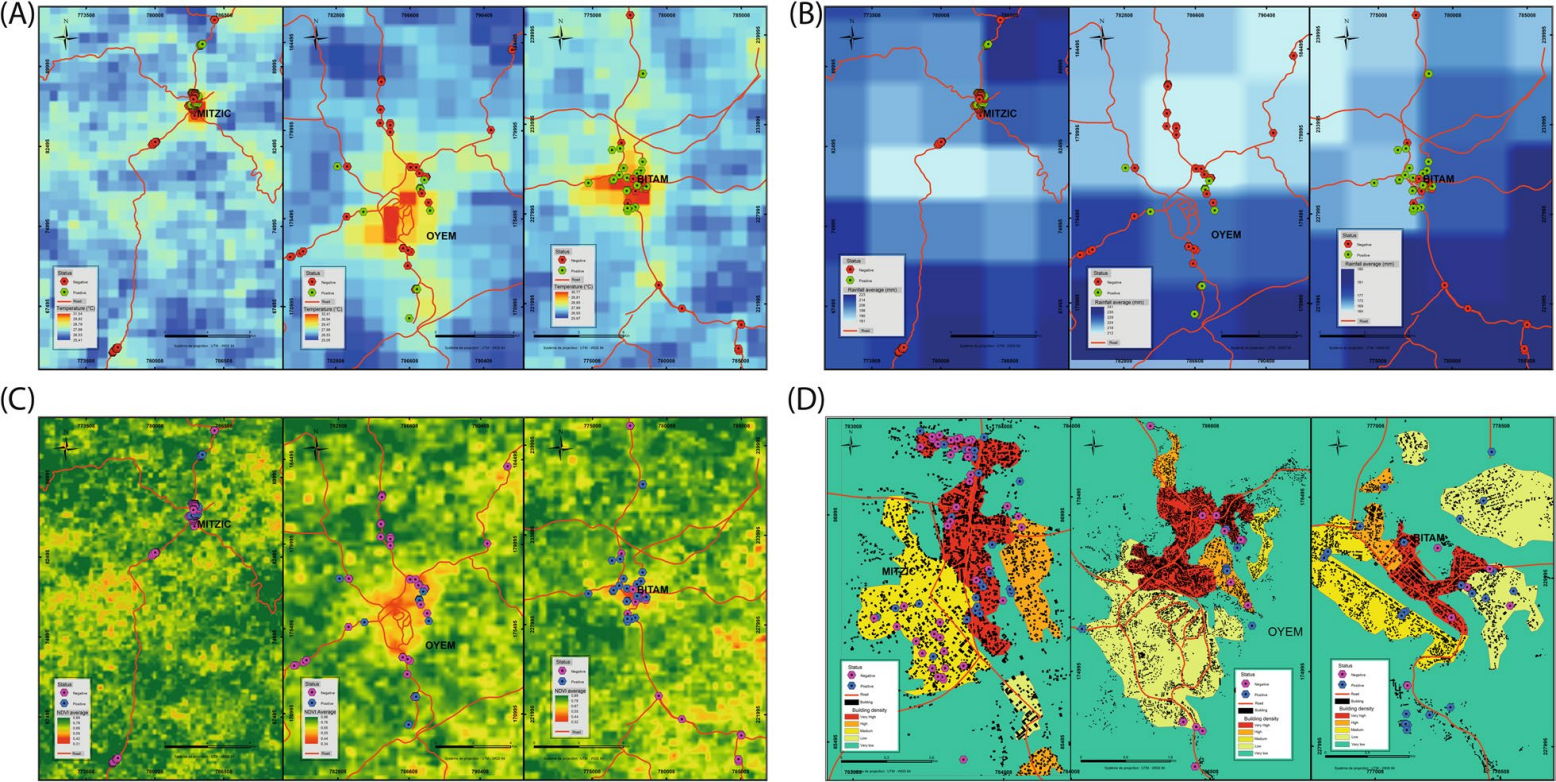
**A** Temperature, **B** Rainfall, **C** Vegetation index **D** Building index and *Anopheles* larval habitats presence: map visual inspection

Geospatial analysis has indicated that temperature, rainfall, vegetation index, and building density have a significant impact on the occurrence of *An. gambiae s.l.* larvae within larval habitats. Specifically, the larval habitats for larvae were notably abundant in high-temperature areas of each town (Fig. 4A) in areas with light precipitation (Fig. 4B). In relation to land use, a medium value of NDVI associated with positive *Anopheles* larval habitats (Fig. 4C) characterized by a high density of buildings (Fig. 4D).

## Discussion

This study aimed to identify and describe the larval habitats of *Anopheles* mosquito larvae in three major towns located in the forest of Northern of Woleu Ntem Province of Gabon. This study used weather and land cover data obtained through remote sensing to create models that to identify regions conducive to the formation of larval habitats. The research is based on the concept of larval source management (LSM), which involves the targeted management of mosquito larval habitats to reduce the population of mosquito larvae. This approach aims to control the spread of mosquito-borne diseases [43, 44]. In recent years, there has been a remarkable expansion of larviciding programmes in Africa, with the aim of eliminating malaria-carrying mosquitoes through ecologically sound methods [8, 9, 45]. However, the achievement of this technique, which supplements LLINs and IRS, will require accurate identification of the multiple larval habitats positive for *Anopheles* and the associated influencing factors. This study provides a basis for this approach in the three main towns of Woleu-Ntem Province, where malaria remains the leading cause of hospitalization [4].

The results shows an overall prevalence of *Anopheles* larval habitats of 33.1% for the three towns, with a significant predominance in Bitam, the least urbanized town in this study. This finding is consistent with what has been observed in numerous studies in sub-Saharan Africa (SSA), indicating that the level of urbanization has a negative impact on the number of *Anopheles* larval habitats [46]. This trend has already been explained by the characteristics of the urban environment, including multifactorial pollution, which makes surface waters less conducive to *Anopheles* aquatic life [47]. Nevertheless, it is important to note that an increasing number of studies conducted in urban contexts in SSA report the presence of *Anopheles* larvae belonging to the *An. gambiae* complex [48–50]. These larvae thrive in larval habitats that were once considered unsuitable for their development due to pollution [47, 51]. This observation might explain why all of the *Anopheles* larvae collected from the larval habitats during the study belong to the *An. gambiae* complex. Species within this complex are known for their remarkable phenotypic plasticity, which allows them to colonize a wide range of larval habitats, as observed in the present study [52, 53]. Satellite data analyses indicate that larval habitats of *An. gambiae* complex species are commonly found in heavily urbanized areas. This can be attributed to the rapid and uncontrolled urbanization in Sub-Saharan Africa, which is characterized by significant rural-to-urban migration, rapid population growth, and informal urban development with precarious installations and inadequate infrastructure [54, 55]. Consequently, rapid and uncontrolled urbanization in sub-Saharan Africa significantly impacts *Anopheles* larval habitats and malaria transmission by creating favourable breeding habitats, increasing the proximity of larval habitats to human dwellings, and potentially contributing to the adaptation of anopheline species to urban ecosystems [46].

Many researchers have investigated the factors that influence the colonization of larval habitats by *Anopheles* mosquitoes, with a particular focus on species within the *An. gambiae* complex [50, 53, 56]. The generalized linear model findings indicate that the presence of *An. gambiae s.l.* larvae in larval habitats is influenced by several factors. At the micro-environmental scale, our study has highlighted the strong association of species of *An. gambiae* complex for ruts and large pools of water. This association is consistent with observations from numerous entomological studies of mosquitoes in urban environments, which have emphasized that larger *Anopheles* breeding habitats, characterized by temporary or semi-temporary lifespans, are mainly associated with ruts and puddles [50, 53, 57]. This preference can be explained by the fact that larger breeding habitats provide more food resources and space for larval movement, thereby offering effective protection against predators such as larvivorous aquatic insects. Furthermore, ecological model highlights that the presence of larvae of certain mosquito species belonging to the subfamily *Culicinae*, such as *Culex* and *Aedes*, acts as a deterrent to the colonization of larval habitats by *An. gambiae s.l.* mosquitoes [58], although positive larval habitats for both *Anophelinae* and *Culicinae* were described. This observation may be due to the various competitive interactions between mosquito species that might affect life history traits, as observed in other studies [59, 60]. Furthermore, despite the increasing occurrence of *Culicinae* larvae in the presence of *Anophelinae* larvae, especially *Anopheles coluzzii* [53], laboratory and field observations clearly indicate that *Culex* spp. larvae remain by far the most adapted to urban pollution conditions [61]. This ability to adapt to environments drastically altered by human activity has led to *Culex* mosquito being the most common mosquito found in large African cities [62, 63]. Although no *An. stephensi* were found during this larval survey, there is a need for such surveys to be repeated especially at/around points of entries of goods which have been identified as the entrance for invasive species in countries through international trade.

At the macro-environmental level, the analysis of satellite data collected during the study period confirmed the influence of climatic factors, such as temperature, rainfall, land use, vegetation index and building density, on the occurrence of *An. gambiae s.l.* larvae in these habitats [64]. In terms of climate, the results showed that *Anopheles* larval habitats were preferentially located in urban hot spots with moderate rainfall. These observations, although similar to those in the 2017 study [65], differ from laboratory results that showed the effect of increased ambient temperature on *Anopheles* life-history parameters such as survival [66, 67] and larval size [68]. This discrepancy in results may be because, unlike in the laboratory where temperature conditions are strictly controlled, ambient temperature conditions in the field are influenced by complex interactions with other factors, such as humidity [69]. In addition, as widely described in literature, rainfall contributes to the formation of temporary or semi-temporary water bodies whose characteristics meet certain requirements of *Anopheles* mosquitoes in the colonization of their larval habitats [70]. In terms of land use, a parameter that is strongly modulated by human activities, the current study highlights the important role played by the vegetation index and building density in the creation of larval habitats favourable to the development of *Anopheles*.

The results showed that larval habitats positive for the *An. gambiae s.l.* were preferentially located at average vegetation index values with a relatively high building density. Their influence on the establishment of *Anopheles* larval habitats is well documented in literature [21]. More importantly, these results reflect anthropogenic activity, which is one of the key factors in the creation of larval habitats favourable to the development of mosquitoes and, in particular, species of the *An. gambiae* complex [65, 71]. Given their crucial role in the colonization of *Anopheles* mosquito larval habitats [53, 57], the absence of water chemistry measurements in this study could be considered as a limitation. Also, it would have been interesting to quantify the *An. gambiae* larval density and correlate it with the environmental and physical parameters considered in the present study to clearly predict the malaria transmission risk in Woleu Ntem province of Gabon. Furthermore, visiting each larval habitat only once does not account for temporal variability. Environmental conditions, such as precipitation, can change over time, which can impact the presence of *Anopheles* larvae. By visiting habitats only once, there is a risk of overlooking this variability, which could compromise the understanding of *Anopheles* population dynamics [72].

## Conclusion

The presence of *An. gambiae* complex larvae in urban larval habitats in the major towns of Woleu-Ntem province is affected by various micro and macro-environmental factors and their interactions, as revealed by this study. These factors include the size of the larval habitat, the presence of other mosquito larvae (micro), temperature, rainfall, land use, vegetation index and building density (macro). Understanding these factors is crucial for the development of effective malaria control strategies, as it allows the targeting of larval habitats that are most conducive to *Anopheles* reproduction, thereby helping to reduce malaria transmission by reducing *Anopheles* population density. Furthermore, larval habitat mapping plays a crucial role in the implementation of these effective anti-vector strategies. In addition, remote sensing is proving to be a valuable tool in the study and management of mosquito larval habitats, which can contribute to the prevention and control of mosquito-borne diseases, particularly malaria. The integration of this tool could facilitate the nationwide characterization of areas potentially favourable for the establishment of *Anopheles* larval habitats. Using ecological modelling tools, it would also be possible to predict the associated risks in these areas, further strengthening malaria control efforts in these malaria hyper-endemic regions.

## Data Availability

The data is available on request through the corresponding author.
